# Single Bacteria Movement Tracking by Online Microscopy – A Proof of Concept Study

**DOI:** 10.1371/journal.pone.0122531

**Published:** 2015-04-07

**Authors:** Andreas Ziegler, Daniel Schock-Kusch, Dominik Bopp, Sandra Dounia, Matthias Rädle, Ulf Stahl

**Affiliations:** 1 Institute for Process Control and Innovative Energy Conversion, Mannheim University of Applied Sciences, Mannheim, Germany; 2 Department of Applied and Molecular Microbiology, Berlin University of Technology, Berlin, Germany; 3 Research Institute for Special Microbiology, Research and Teaching Institute for Brewing in Berlin, Berlin, Germany; Loyola University Chicago, UNITED STATES

## Abstract

In this technical report we demonstrate a low-cost online unit allowing movement tracking of flagellated bacteria on a single-cell level during fermentation processes. The system’s ability to distinguish different metabolic states (viability) of bacteria by movement velocity was investigated. A flow-through cuvette with automatically adjustable layer thickness was developed. The cuvette can be used with most commercially available laboratory microscopes equipped with 40× amplification and a digital camera. In addition, an automated sample preparation unit and a software module was developed measuring size, moved distance, and speed of bacteria. In a proof of principle study the movement velocities of *Bacillus amyloliquefaciens* FZB42 during three batch fermentation processes were investigated. In this process the bacteria went through different metabolic states, vegetative growth, diauxic shift, vegetative growth after diauxic shift, and sporulation. It was shown that the movement velocities during the different metabolic states significantly differ from each other. Therefore, the described setup has the potential to be used as a bacteria viability monitoring tool. In contrast to some other techniques, such as electro-optical techniques, this method can even be used in turbid production media.

## Introduction

Due to the pressure by regulation agencies like the US Food and Drug Administration on more sophisticated process control and documentation in fermentation processes an effort was put in recent years towards the development of novel real time monitoring systems [1, 2]. A major requirement of process control systems is that data be available in real-time in order to intervene control as soon as possible in case of inadvertent events, such as nutrient limitation, contamination by phages or overgrowth by unwanted strains. Moreover the systems are desired to be independent of sampling to keep the sterile borders of the fermentation vessel closed during the whole process.

Several online and atline optical, spectroscopic, and capacitive probes and techniques were developed focusing on cell density or excreted metabolites in this respect [1–5]. In contrast, less effort has been put towards techniques allowing the monitoring of physiological parameters like cell viability [1]. The highly sensitive “gold standard” approach for cell viability testing are still plate count methods [6–8]. However, as they rely on incubating samples outside the bioreactor on nutrient agar they do not match the above mentioned requirements of process control systems.

Also, flow cytometry is frequently used for cell viability testing. Results can be achieved within 20 min after sampling, depending on the staining process used [9–12]. Also fully automated combined cytometry and bioreactor systems are described [13–15]. However, complicated handling and high cost of flow cytometry systems prevent them from being used as standard procedure.

Other methods for cell viability measurement are based on online real-time electro-optical measurements, such as the EloTrace (EloSystems, Berlin) [16–18], or inline optical methods such as focus beam reflection probes (FBRM) [2] and the recently published three dimensional optical reference method (3D-ORM). In contrast to the FBRM probe, the 3D-ORM probe also allows measurements of spherical bacteria [1]. There are also other 2D and 3D bacteria movement tracking systems described in literature [19–26]. In contrast to the one presented none of these combine all requirements on on-line systems, namely: automated sampling, automated data acquisition and automated movement tracking in real time.

In Table 1 current methods for viability testing with their pros and cons are summarized.

**Table 1:** Comparison of current viability measurement techniques.

Method	Measured parameter	Intervall	+ advantage	- disadvantage
plate count	ability of cell division on nutrient plates; detection of colony forming units (CFU)	offline	well established; cell division often the most important parameter	offline method-no detection of VBNCs (viable but not culturable); long incubation times required
Flow Cytometry / FACS	vital staining; intercalation of dyes in DNA of living and dead cells	ca. 20 min	well established; detection of VBNC; cell sorting; High-Throughput-Screening	high costs
classic staining and microscopy	staining capability of the cell and cell components	offline	well established; detection of VBNC	offline method; complex sample preparation
metabolic activity (ATP content)	staining and detection of living cells	offline	well established assays available	total cell count is needed
physiologic activity (e.g.HPLC, pH, pO_2_)	metabolism	offline; online	cheap; well established probes available	process parameter have to be well known for data interpretation
electrooptical measurement	polarizability	ca. 20 min	automated sample preparation and data analysis	not possible in turbid media; only rod shaped bacteria can be measured; high costs
here: automated movement tracking	motility	ca. 20 min	clear parameter; measurement in turbid media possible; automated sample preparation and data analysis	possible with flagellated cells only; process parameter have to be well known for data interpretation

One aspect not addressed by available probes, for monitoring cell viability, is cell motility. Grossart et al. [27] could show, by incubating sea water bacteria in high glucose or peptone media, that motility of the motile bacteria fraction increased when grown in nutrient excess. They proposed that motility may serve as an indicator for viability of bacteria, as motility requires high amounts of energy. Therefore, only bacteria with high nutrition and health status will move.

In this technical note we demonstrate a low cost, online unit allowing movement tracking of flagellated bacteria at the single cell level. The system was tested during batch fermentations of *Bacillus amyloliquefaciens* FZB42 with well-known growth characteristics. Its ability to distinguish different metabolic states (viability before, during and after diauxic shifts as well as during onset of sporulation) of the bacteria by movement velocity was investigated. Therefore a flow through cuvette with adjustable layer thickness was developed, preventing the bacteria from leaving the optical plane along the z-direction. The cuvette can be used with most commercially available laboratory microscopes equipped with 40x amplification and a digital camera. In addition to the automated sample preparation unit, a software module was developed measuring size, distance travelled and speed of bacteria detected in the optical plane.

Flagellated-Bacteria lose their ability to move when they are exposed to high shear stress due to deflagellation [28]. Therefore, shearing of flagella during the sampling procedure must be avoided for accurate measurement. The influence of shear stress on the motility of *Bacillus amyloliquefaciens* FZB42 was investigated and maximal possible shear stress in the system calculated.

## Materials and Methods

**Fig 1:**
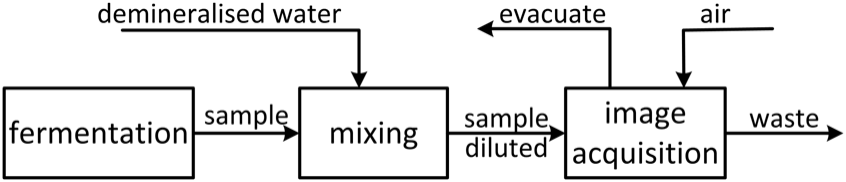
Simplified process flow diagram of the sample preparation and image acquisition. The sample mixed with demineralized water is pumped through the flow cell. After evacuation the image acquisition can start. Before the next sampling aeration and disposal is necessary.

In this study a low cost online unit allowing movement tracking of flagellated bacteria on single cell level during fermentation processes was developed. Fig. 1 depicts the simplified process flow diagram of the set up.

The sampling (approx. 40 ml) from the fermenter is realized with a small dead volume (approx. 0.4 ml, approx. 1% of sampling) via a valve. Depending on the fermentation stage, the sample is diluted with demineralized water. This sample preparation is necessary to reduce the number of bacteria per image (optimum between 5 and 10). The diluted sample is pumped through the flow cell. By vacuum, the layer thickness is minimized in the flow cell making the imaging possible. After aeration and disposal a subsequent sampling can start.

### Automated sample preparation

**Fig 2:**
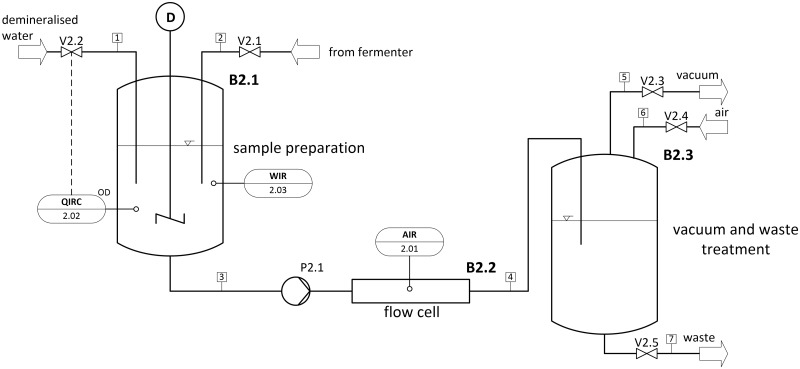
Simplified flow chart of the sample preparation, image acquisition, vacuum and waste treatment. The measurement of OD at 600 nm (QIRC 2.02) regulates the dilution of the sample (stream 1). The degree of sample dilution is registered using weight control (WIR 2.03). In the flow cell, the image acquisition (AIR 2.01) can start after evacuating the system.

Sample preparation is accomplished by real-time measurement of the optical density (OD) at 600 nm of the samples taken. Depending on the fermentation phase, the samples have to be diluted with demineralized water to an OD of 1.15. The OD of 1.15 was found to be optimal for the following movement investigations in the flow cell in preliminary experiments. The optimum OD depends on the nutrient solution and the type of bacteria cultivated. For other cultivations, optimal OD has to be adjusted. With more than 15 viable bacteria the automated movement tracking becomes inaccurate. The degree of sample dilution is determined and monitored gravimetrically by automated weight control of the sample (Ohaus, Adventurer Pro). Fig. 2 illustrates the simplified flowchart of the developed unit, consisting of the sample preparation unit, the flow cell, vacuum pump and waste treatment.

### Flow cell with adjustable layer thickness

**Fig 3:**
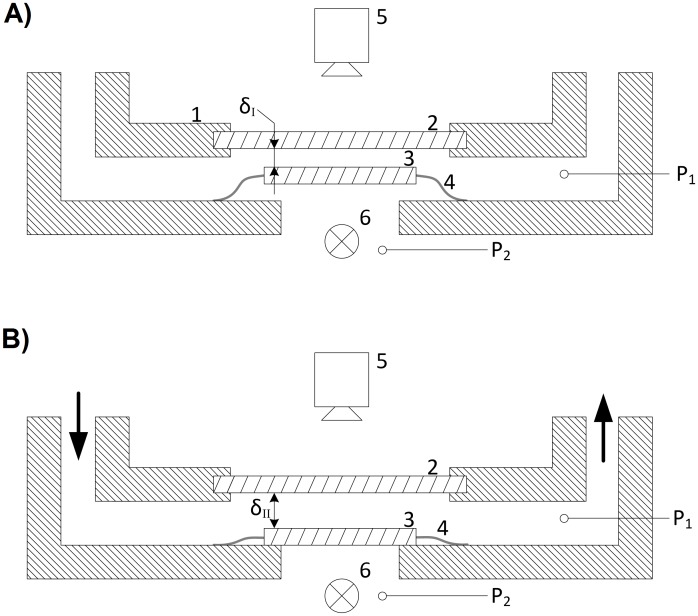
Schematic representation of the operating principle of the flow cell. A) P1 < P2, δI < 5 μm, ready for image acquisition. B) P1 > P2, δII > 1.2 mm. 1: housing 2: cover slip 3: microscope slide 4: membrane 5: image acquisition 6: illumination.

The core of the system is the novel flow cell with adjustable layer thickness (Fig. 3). It consists of a silicone membrane with an integrated polycarbonate microscope slide. When the sampling pump (P2.1) is started, the silicone membrane inflates automatically as P_1_ > P_2_. Wide diameters of inlet and outlet and a wide layer thickness of the flow cell (δ_II_ > 1.2 mm) prevent destruction of flagella of the bacteria by shear force. Low pressure and low shear force at flow rates of 30–60 ml min^-1^ in the cuvette are only possible with a wide layer thickness. Automatically the layer thickness δ_I_ is adjusted to have a maximum thickness of 5 μm within 30–60 seconds after connecting the vacuum when P_1_ < P_2_. The remaining fluid movement is low and doesn’t influence the movement tracing of bacteria.

### Data acquisition

The cuvette is placed under a microscope equipped with a 40 fold amplification phase contrast objective lens with a numerical aperture of 0.65 (Olympus, PLCN40XPH). Image acquisition was accomplished using a GigE-color camera DFK23G445 (Imaging Source) with a resolution of 1280 x 960 pixel and a frame rate of 30 fsp. With these settings one pixel equals a square of 94.2 nm edge length, creating an imaging region of 120.6 x 90.4 μm. The layer thickness of max. 5 μm prevents bacteria from leaving the depth of field of the objective lens in the z direction, thereby allowing movement tracking throughout the entire field of view.

### Software based automated single bacteria tracking

The tracking software was programmed in NI LabVIEW 2013 with the NI LabVIEW Vision Development Module.

**Fig 4:**
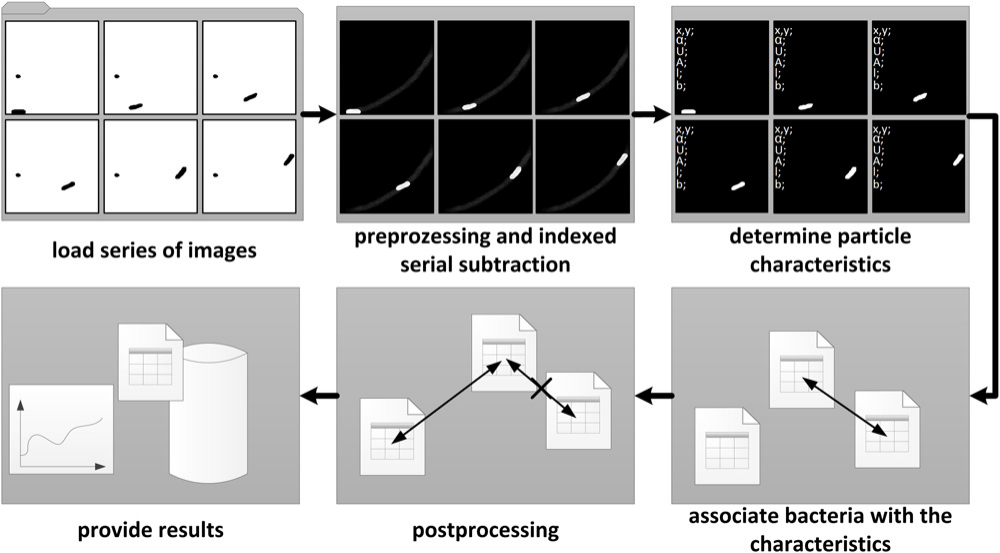
Simplified software structure of the image analysis. First, the series of images (30 frames at 30 fps represents 1 s) are loaded, before the indexed serial subtraction and the image processing starts. Now particle characteristics (center of mass x, center of mass y, perimeter, area, orientation, length, width) are determined. In the next step the bacteria are associated with the characteristics. After post-processing the results are provided.

The single bacteria tracking software allows the automated differentiation between particles and bacteria, counting bacteria, tracking the movement of the identified bacteria, determining the speed of movement and the determination of size and shape of the bacteria. A simplified structure of the software is shown in Fig. 4.

The program is optimized for processing picture series of 1280 x 960 pixel in PNG format. Pictures can be uploaded manually or can be automatically analyzed immediately after acquisition of the series. The software requires the original pictures from the camera without preprocessing. During the whole process the original pictures are saved. The evaluation time of one series of photographs on a standard Windows PC, depending on the quality of the images and the number of detected viable bacteria takes 1–2 minutes.

**Fig 5:**
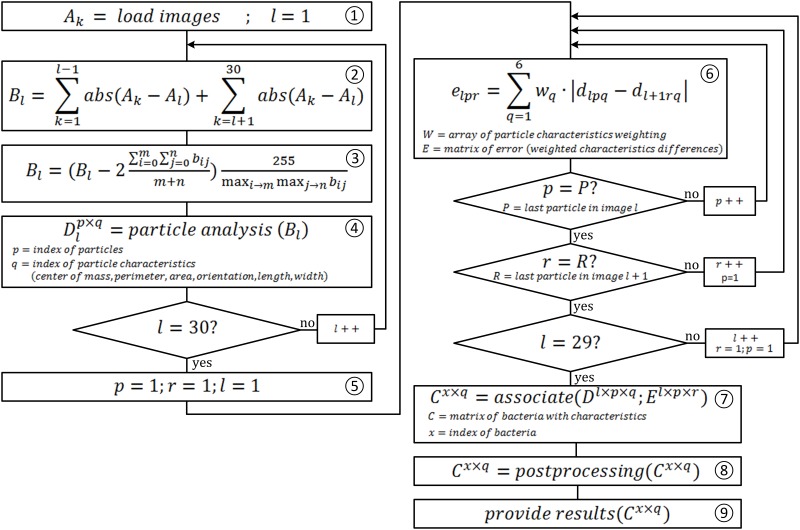
Simplified program flow chart of the image analysis. Simplified program flow chart of the image analysis. In step 1 the images are loaded, step 2 shows the indexed serial subtraction, step 3 the image preprocessing in step 4 the particle characteristics are determined, step 6 and 7 association of the bacteria with the characteristics, in step 8 the assignment is optimized before results are provided in step 9.

For a better understanding of the simplified program flowchart (Fig. 5), the following definitions are necessary.

The matrix space formed by the triple [*M*,+,·] over the field R^*m*×*n*^, with the set *M*, the function component wise addition

$$\begin{array}{c} \langle +\rangle \;:ℳ\times ℳ\to ℳ\;\;\;\;\\ \left(A,B\right)\;\mapsto A+B=\left(a_{ij}+b_{ij}\right) \end{array}$$

and the function component wise scalar multiplication

$$\begin{array}{c} \langle \cdot \rangle \;:\mathbb{R}\times ℳ\to ℳ\;\;\;\;\\ \left(\lambda ,A\right)\;\mapsto \lambda \cdot A=\left(\lambda \cdot a_{ij}\right)\;\;. \end{array}$$

In addition, the component wise absolute value is defined as

$$\begin{array}{c} \langle abs\rangle \;:ℳ\to ℳ\;\;\;\;\\ \left(A\right)\;\mapsto abs\left(A\right)=\left(\left|a_{ij}\right|\right)\;\;. \end{array}$$

The digital images (matrices) *A*, *B* ∈ *M* with the resolution (dimensions) *m*×*n* consist of the picture elements (components) *a*_*ij*_ *b*_*ij*._

Following the simplified program flowchart (Fig. 5) the automated image analysis is described in more detail. The program flowchart in Fig. 5 was simplified to its essentials to make it easier for the reader to understand. Steps such as conversion of color image into a grayscale image, normalizing the particle characteristics for the weighting, the function of removing small particles or the scaling of the pixel space in the metric space are not illustrated.

In step 1, the image series, consisting of 30 individual images, *A* is loaded. From these 30 images *A* 30 sum-difference images *B* are generated in step 2 by indexed serial subtraction. The current image formed by computing the component-wise subtractian with the 29 other pictures in the series. Following the component-wise sum of the absolute value of the 29 difference images is calculated. From the individual results, a sum-difference image is generated by a component-wise addition. In step 3, using the sum-difference image, the image noise is removed and the contrast optimized. The two-time average of the pixels is subtracted to remove image noise. The contrast is then normalized to have values from 0 to 255. The Equation (Fig. 5, Step 3) would also create negative pixel values, however these are set to zero due to the data type of the image *B*. The function shown as "particle analysis" in step 4 is based on the subprogram "IMAQ Particle Analysis" of the LabVIEW NI Vision Development Module. The function "particle analysis" detects several particles for each sum-difference-image(*B*). These detected particles have the following characteristics: center of mass X, center of mass Y, perimeter, area, orientation, length and width. These characteristics are stored in the particle-matrix(*D*). In step 6, which is located in a triple loop, the sum of weighted characteristic differences are formed of all particles found in two sequential images. The differences in characteristics are stored in the error-matrix (*E*). Based on the error-matrix *E* and particle-matrix *D*, the assignment of the individual particles to each other is implemented. The function "associate" in step 7 represents a complex association algorithm based on the smallest particle characteristic differences and stores the results in the bacterial-matrix C. In this step the core of the single bacteria tracking is implemented. The algorithm prevents double assignments if two bacteria cross and recognizes the disappearance of bacteria from the image area. In step 8, the "post-processing", several functions are summarized like the scaling of pixel space in the metric space. Furthermore, the assignments of the particles to be checked in terms of quality and validity are accomplished in step 8. If the recording quality, due to high content of suspended solids, artifacts or an excessive bacteria concentration, is insufficient, gaps or errors in the single bacterial tracking can occur. Gaps are closed here, and incorrect data records removed. In the final step, step 9, the results are provided, including the compression and visualization of the data as well as export opportunities of these records.

### Media and culture conditions

For batch fermentation, 9 l distilled H_2_O, 85,5 g complex production media (ABiTEP Berlin) and 1 ml Aspumit AP anti foaming agent (Thor GmbH Speyer) was filled in a 14-l fermenter (Chemap AG, Typ: 9–2703). PH was adjusted to 7.2 using 5% KOH (66,5 ml). Media was sterilized at 121°C for 30 min in the fermentation vessel. After the sterilization process, aeration was started (100 ml min^-1^) and media cooled to 80°C. Following, the media was inoculated with 1.35 g *Bacillus amyloliquefaciens* FZB42 [29–31] spores, 4×10^11^ cfu g^-1^ (ABiTEP Berlin). The Strain FZB42 can be obtained from the Leibniz Institut DSMZ-German Collection of Microorganisms and Cell Cultures under the DSM No.: 23117. Temperature was maintained at 80°C for 30 min before the media was cooled down and maintained at 35°C. pO_2_ was automatically regulated to 50% by the agitation speed (min. 225 rpm) and the aeration (min. 100 ml min^-1^). During the fermentation process pH (405-DPAS-SC-K82/325, Mettler Toledo), pO2 (InPro 6820/12/320 Mettler Toledo), agitation and aeration (Brooks, Mass-Flow-Controler 5850E) were constantly monitored in line. For viability, measurement samples were automatically taken (max 40 mL sample^-1^) periodically (about every 20min) starting 15 min after the media reached 35°C.

Bacteria velocity data was evaluated for normal distribution by Shapiro-Wilks, Anderson-Darling and Cramér-von Mises tests. Evaluation for significant differences in bacteria velocity at different metabolic states was performed by Mann-Whitney U test since velocity data was found not to be normally distributed.

Automated image analysis data was validated at the four stages of interest (before diauxic shift, diauxic shift, after diauxic shift, start of sporulation) by manual evaluation of the image series for fermentation 1. More than 7500 individual velocities had to be extracted by hand for the comparison. The centroid of the bacteria was marked by hand and then the individual velocities from the euclidean distance determined.

### Influence of shear stress

Deflagellation due to shear stress by the sampling and pumping procedure would lead to faulty results of the measurement.

To determine the critical shear stress leading to deflagellation of *Bacillus amyloliquefaciens* FZB42 samples were pumped through defined capillaries. To prevent clotting of capillaries the bacteria were cultured in 6.25 g l^-1^ “Standard Nutrient Broth I” (Carl Roth GmbH + Co. KG) and samples drawn in the exponential growth phase. All other fermentation parameters were kept as described in “Media and culture conditions”. Three 20 ml samples were filled in syringes (Injekt 20 ml; Luer Solo, B.Braun Medical) and simultaneously pumped through three capillaries by a syringe pump (Fusion 400, Chemyx Inc., USA). The flow rate was 4 ml min^-1^. The three capillaries were each 0.6 m long. The inside diameter of the capillaries were 1 mm, 0.5 mm and 0.25 mm respectively. 15 samples were measured in each capillary. Immediately after the treatment, a series of images for motility assessment was assessed. The samples were placed on a slide and examined with a video-microscope (CX31, Olympus Corporation, Japan). The image acquisition was carried out in transmitted light with a 20x objective (PLCN20XPH, Olympus Corporation, Japan). The camera used is a GigE color camera (DFK 23G445, The Imaging Source Europe GmbH). The resolution of the camera was 1280 x 960 pixels at a frame rate of 30 fps. Each pixel consisted of three 8 bit values, 8 bit values for red, green and blue each. The sensor size is 1/3 ". The recorded image series consisted of 15 photographs, this corresponded to 500 ms. To eliminate the influence of the delay time, the order of measurements were reversed (1–0.5–0.25 mm and 0.25–0.5–1 mm). The image data were then analyzed by a LabVIEW (LabVIEW 2013, National Instruments) program. The self-developed LabVIEW program plays the photos from an image series in a loop. Bacteria counting was performed by manual counting. The user can select motile and non-motile bacteria. The proportion of moving cells was then exported. For shear force calculation also the density (pycnometer (Schott AG, Germany) at 35°C) and the viscosity (capillary viscosimeter Typ 530 10 / I (SI Analytics GmbH, Germany) at 35°C) of the media was assessed.

Shear force calculation was performed using the „narrow channel” assumption by Herwig [32] under laminar flow conditions [33].

Taking these assumption into account the shear force in the capillaries and the tubing of the system can be calculated [32,33]. The velocity profile $\frac{u}{u_{m}}$, is defined as:

uum = 2 1-yD22

Where *u* is the velocity at a position *y* and *u*_*m*_ the velocity averaged on the capillary diameter (D) The velocity gradient $\dot{\gamma }$ can be calculated by the velocity change in *y*-direction:

$$\dot{\gamma }\;=\;\frac{\Delta u}{\Delta y}\;=\;\frac{du}{dy}$$

The shear stress τ can be assessed as shear force *F* over area *A*. The dynamic viscosity *η* multiplied by the shear stress results in the in the shear stress.

$$\tau \;=\;\eta ∙\dot{\gamma }$$

The Reynolds Number *Re* allowes a statement about the laminar or turbulent behaviour of the stream in a pipe. When *Re* is < 2300 laminar behavior can be assumed [33]. Using the kinematic viscosity *v*, *Re* can be calculated as.

$$Re\;=\;\frac{u_{m}\;D}{\nu }$$

The statistic evaluations were performed using R. Samples were tested for normal distribution using Shapiro-Wilk-Test, Anderson-Darling-Test and Cramér-von-Mises-Test, moreover the data was evaluated using T-Test and F-Test.

## Results and Discussion

Major aim of the study was the investigation if the developed system for online bacteria movement tracking has the ability to measure changes in movement velocities of flagellated bacteria during fermentations. The system was tested during *Bacillus amyloliquefaciens* FZB42 batch fermentations with well-known growth characteristics. Its ability to distinguish different metabolic states (viability before, during and after diauxic shift as well as during onset of sporulation) of the bacteria by movement velocity was investigated. As validation parameter for the different metabolic states in this study the characteristic pH profile (Diauxic shift at pH minimum) and growth rate of measured motile cells were chosen.

Cell viability is a crucial parameter for quality control of fermentation processes. Current methods are either time intensive (plate counting), cost intensive (flow cytometry) or cannot be used in turbid production media, such as probes based on polarisability (Elo-Trace). Based on the findings of Grossart et al. [27], that motility of bacteria rises when incubated in nutrient rich media and motility therefore might be a reasonable parameter for viability; a fully automated technique allowing motility monitoring of flagellated bacteria during fermentation was developed.

**Fig 6:**
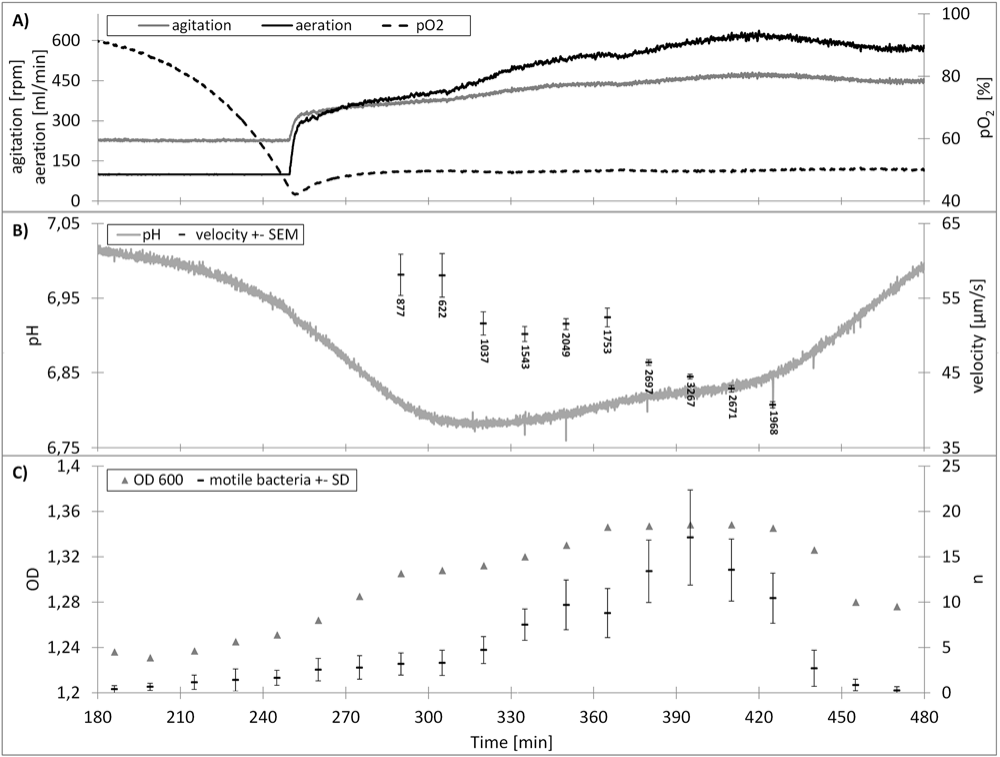
Cultivation of *Bacillus amyloliquefaciens* FZB42. A) Monitored aeritation and agitation data. B) pH and mean bacteria velocity, below the bars are the numbers n of determined velocity vectors per sample. C) optical density (OD 600) and mean number of motile bacteria observed in the field of view of the flow cell, corrected by dilution factor of the sample.

The online system developed consists of a sample preparation unit, flow cell, vacuum pump, waste treatment and software allowing movement tracking of bacteria at the single cell level. Besides movement tracking, the system also allows observation of cell size and shape and a relative measure of cell density of motile bacteria through analysis of the required dilution of the samples and the number of observed bacterium per image (see Fig. 6C). Automated sample extraction, image acquisition and data analysis per sample takes less than 5 minutes, thereby allowing fermentation monitoring in nearly real time. Using the current optical setup cells of a min. diameter of 0.4 μm can be detected. However, it is possible using an objective with higher amplification (eg. 100x), which would allow the detection of cells with 0.2 μm.

For the proof of concept, *Bacillus amyloliquefacien* FZB42 fed batch fermentations were used. *Bacillus amyloliquefacien* FZB42 is a strain known for extensive sporulation. The spores are commercially used as fertilizer (RhizoVital 42, ABiTEP) [34]. The strain has been well studied in terms of its plant growth-promoting properties.

In the vegetative growth phase (nutrition excess), *Bacillus amyloliquefacien* FZB42 produces organic acids through starch metabolization, leading to a drop in pH in the fermentation vessel. When all nutrients are metabolized, *Bacillus amyloliquefacien* FZB42 shifts its metabolism by a diauxic shift to organic acid consumption, leading to a rise in pH. After organic acid consumption, sporulation starts.

Fig. 6 depicts exemplary monitoring data of a *Bacillus amyloliquefaciens* FZB42 fermentation. With increasing cell density at min. aeration (100 ml min^-1^) and min. agitation (225 rpm) the pO2 drops. By controlling the aeration and agitation the pO2 is kept above 50% throughout the whole process (Fig. 6A). Fig. 6B depicts the pH levels with an expected drop due to acid production of the *Bacillus amyloliquefaciens* FZB42 before a rise in pH was observed due to the known diauxic shift of the *Bacillus amyloliquefaciens* FZB42 associated with metabolisation of the produced organic acids. The bacteria velocity data shows a drop after the pH reached its minimum and rises again after the diauxic shift is completed, before dropping again when sporulation starts. In Fig. 6C the OD 600 measurement is shown with the mean number of bacteria observed in the single images of each series of 30 images.

Monitoring of the bacteria motility did show the same trend during the fermentation process in all three fermentations (Table 2). A drop in movement velocity during the diauxic shift, at pH minimum followed by a rise of the velocity during organic acid metabolization (when pH rises again), before another velocity drop during sporulation (drop in OD 600).

**Table 2:** Summary of the specific stages of the three monitored fermentations.

		Ferm. 1	Ferm. 2	Ferm. 3
before diauxic shift	mean velocity ± SEM (μm s^-1^)	**50.74** ± 1.34	**51.03** ± 2.11	**58.04** ± 2.92
	Number of velocity vectors	210	675	622
	Measurement time (min)	232.00	304.00	305.00
	mean bacteria per image	1.07	2.43	3.31
	pH	6.85	6.78	6.79
diauxic shift (minum pH)	mean velocity ± SEM (μm s^-1^)	**47.71** ± 0.64	**46.17** ± 0.82	**50.17** ± 1.01
	Number of velocity vectors	673	636	1543
	Measurement time (min)	292.00	338.00	335.00
	mean bacteria per image	5.95	2.90	7.53
	pH	6.68	6.77	6.79
after diauxic shift	mean velocity ± SEM (μm s^-1^)	**51.88** ± 0.37	**59.56** ± 0.39	**52.39** ± 1.26
	Number of velocity vectors	2820	3182	1753
	Measurement time (min)	352.00	360.00	365.00
	mean bacteria per image	13.69	14.57	8.80
	pH	6.75	6.80	6.81
start of sporulation	mean velocity ± SEM (μm s^-1^)	**46.16** ± 0.44	**44.95** ± 0.38	**40.76** ± 0.41
	Number of velocity vectors	1624	2794	1968
	Measurement time (min)	382.00	391.00	425.00
	mean bacteria per image	9.41	13.62	10.43
	pH	6.82	6.84	6.85

Statistical evaluation by Shapiro-Wilks, Anderson-Darling and Cramér-von Mises tests revealed that the measured velocity data per stage are not normally distributed, therefore, a Mann-Whitney U test was used to test if the bacteria velocities at the distinct time points differ significantly. As shown in Table 3, besides comparison before diauxic shift vs. diauxic shift of fermentation 1 the bacteria velocities at the different metabolic states differ significantly. As a sampling rate of about 20 min was chosen, the minimum in mean velocity may have been missed during fermentation 1. However as stated before, sampling rate can be increased to one sample per five minutes.

**Table 3:** Comparison of the specific stages of bacterial growth by Mann-Whitney U test.

	Ferm. 1 p-value	Ferm. 2 p-value	Ferm. 3 p-value
before diauxic shift vs. diauxic shift	0.1120	<0.0001	<0.0001
diauxic shift vs. after diauxic shift	<0.0001	<0.0001	0.0068
diauxic shift vs. start of sporulation	<0.0001	<0.0001	<0.0001

**Fig 7:**
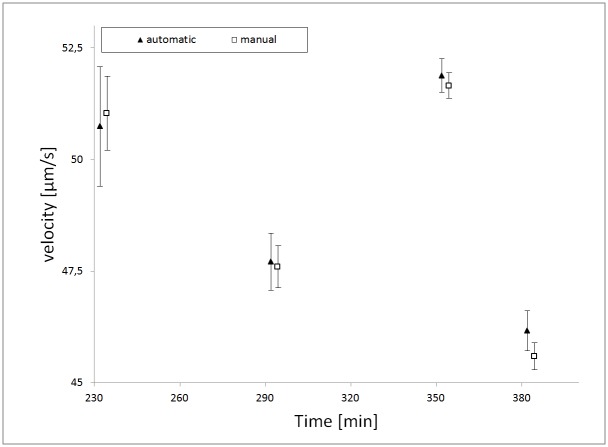
Validation of automatic image analysis. The validation of automatic image analysis by manual analysis of the image series on the four significant sampling points of interest (before diauxic shift, diauxic shift, after diauxic shift, start of sporulation) during fermentation 1. The error bars represent the Standard Error of the Mean of the bacterial velocity. The number of determined velocity vectors per sample are 232: n_man_ = 354, n_aut_ = 210; 292: n_man_ = 1150, n_aut_ = 673; 352: n_352man_ = 3818, n_aut_ = 2820; 382: n_man_ = 2600, n_aut_ = 1624.

To check the bacteria movement data by the software for plausibility, manual movement tracking was performed for fermentation 1. As shown in Fig. 7 a good correlation was found, indicating accuracy of the automated measurements (in contrast to minutes processing time of the automated system, manual movement tracking takes days of manual analysis). Velocity data is also in line with findings by Mendelson et al. [23] and Harshey et al. [35] who measured velocities of *Bacillus subtilis* between 76 and 116 μm s^-1^ and about 40 μm s^-1^ in *Escherichia coli* and *Salmonella typhimurium* respectively.

**Fig 8:**
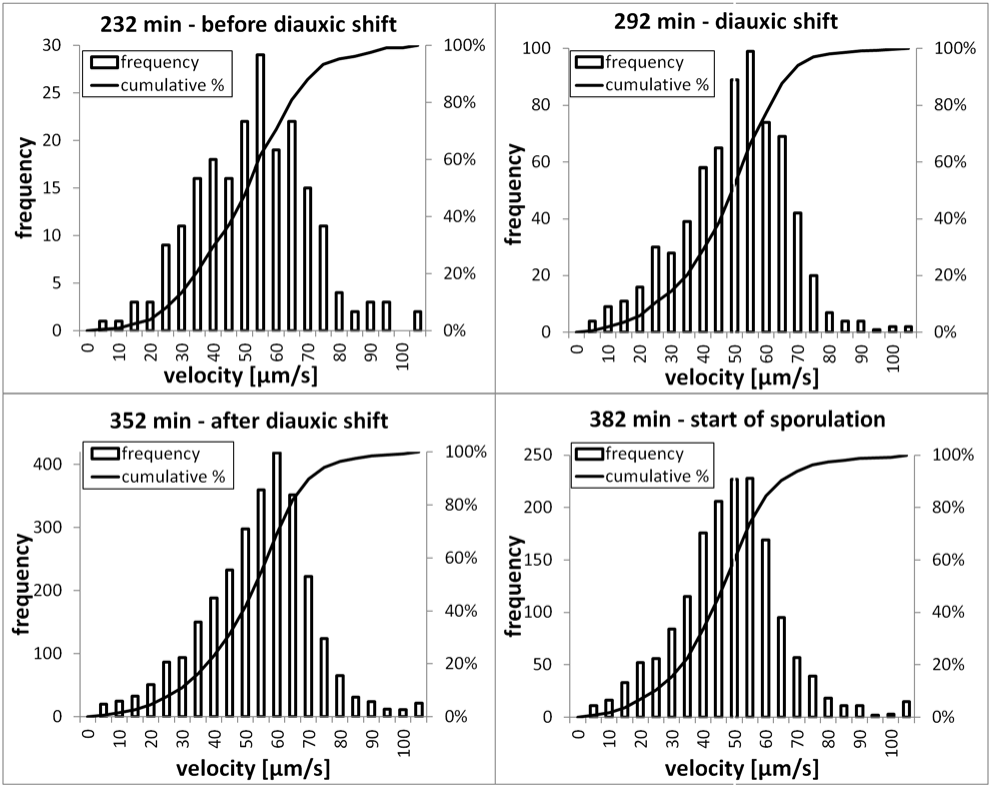
Histograms of individual velocities. The Histograms produced by automatic image analysis on the four significant sampling points of interest (before diauxic shift, diauxic shift, after diauxic shift, start of sporulation) during fermentation 1.

In Fig. 8 the four histograms of the individual velocities of the automatic image analysis during the sampling points of interest of fermentation 1 are shown. Despite statistically significant mean velocities at the different metabolic states the velocity distributions are broad and highly overlapping. Therefore the clear identification of a metabolic state just by one measurement of the velocity at a random time point during fermentation seems to be difficult or even impossible. Also a comparison of the velocities of the bacteria at the different stages in between the fermentations differ significantly (S1 Table). Reasons therefore might be differences in inoculation, nutrient concentration, aeritation or others which accidently occured. This has to be further investigated. Therefore, instead of the overall value of the mean velocity the changes over time of the mean values during fermentation are the parameters of interest to draw conclusions from the measurements.

As shown by Grossart et al. [27] heavy shear force can lead to flagella rupture in bacteria, leading to motility loss. Therefore, it was essential that the system operates at slow flow rates and wide inlet diameter for the flow cell in order to prevent flagella destruction. Furthermore, the adjustable layer thickness of the flow cell is very important to minimize the shear forces. It could be shown that the shear stress occurring in the system does not lead to deflagellation of *Bacillus amyloliquefacien* FZB42. The calculation of the occurring shear stress in the sampling unit and the flow through cuvette resulted in a maximum of 2000 Pa. In the experiments evaluating the motile bacteria fraction, shear stress in a range of about 500–33000 Pa was applied on the Bacteria. No significant difference in the motile bacteria fractions were observed, indicating that no deflagellation occurred (Tables 4 and 5).

**Table 4:** Flow conditions in the capillary of shear stress evaluations.

Inside diameter of the capillary	D = 1 mm	D = 0,5 mm	D = 0,25 mm
Reynolds number	108	216	432
Mean speed u_m_ (mm s^-1^)	84,8	339,5	1.358
maximum speed u_max_ (mm s^-1^)	169,8	679	2.716
maximum shear stresss τ_max_ (Pa)	525,5	4.161,8	33.294
Mean percentage value of motile bacteria fraction ± standard deviation	28.88 ± 12.70%	28.14 ± 11.34%	28.46 ± 13.46%

**Table 5:** Statistical analysis of shear stress evaluations.

	p-value		
	D = 1 mm	D = 0.5 mm	D = 0.25 mm
Shapiro-Wilk normality test	0.08746	0.6425	0.2445
Anderson-Darling normality test	0.2243	0.5279	0.1717
Cramer-von Mises normality test	0.4548	0.4001	0.143
	D = 1 mm and D = 0.5 mm	D = 1 mm and D = 0.25 mm	D = 0.5 mm and D = 0.25 mm
Two Sample t-test	0.9479	0.9337	0.8734
F test to compare two variances	0.4947	0.7863	0.6796

Another aspect that might influence the motility of the bacteria are surface effects in the flow through cell due to its narrow layer thickness. Frymier and Vigeant could show that the mean velocities of bacteria close to surfaces slows down, and the movement pattern change [22, 24]. Though, as the surface effects in the flow through cell are the same for all samples this should not influence the predictive value of the measurement. However, due to the presence of the surface effects the measured velocities and movement pattern in the flow through cell might not reflect the behavior of the bacteria in larger volumes with no impact of surfaces.

An advantage of the system is its usability in turbid production media. As undissolved particles do not move in the flow cell, they are not registered by the software and therefore do not interfere with measurements. Turbid media is especially a problem for viability probes based on polarizability as they rely on an OD measure which is only related to bacteria. Nutrient particles that dissolve during the fermentation process result in a bias of this measurement. The same might be true for probes based on light scattering or reflectance as the particles may lead to artifacts.

A drawback of this method is its sample volume requirement (approx. 40 mL sample^-1^), which may lead to a significant loss of fluid and therefore product during the process if a high sampling rate is chosen. Thus, further development is necessary in order to minimize the required volume. Since the whole system is autoclavable, a recirculation of the sample is possible. However, in batch fermentation an additional sample concentration unit would be necessary (e.g. ultra-filtration unit) to reduce the sample volume back to the initial volume. Also a cleaning step, flushing the system between the samplings, should be introduced to prevent possible accumulations and contamination of the flow cell. However, no excessive accumulation or contamination interfering the measurements occurred in the experiments. The rare particles that contaminated the flow cell generally did not move and therewith did not affect the measurement. Moreover, the system cannot be used for bacteria that are not flagellated.

Most inadvertent events result in slower growth rates and loss of viability of the culture. Our hypothesis is, that such events also affect the velocity of the bacteria. In this study we could show that this hypothesis is true for nutrient limitation leading to diauxic shift or sporulation.

## Conclusions

In summary, this study shows that the developed system can monitor changes in motility of *Bacillus amyloliquefaciens* FZB42 during batch fermentation. These changes in motility correlate with known changes in the metabolic state of the bacteria (validated by pH and OD monitoring). Therefore, this technique might have potential as a viability monitoring system during fermentation processes. Major aim of the study was to investigate if the system has the ability to measure changes in movement velocities of flagellated bacteria. Excessive further research is necessary, investigating motility changes due to other stressors than nutrient limitation (e.g. Infections with Bacteriophages, low and high temperature, low oxygen conditions etc.), and if the observed changes in motility are also present in other relevant production strains as e.g. *Escherichia coli* and *Bacillus subtilis*. Moreover the system has to be validated versus plate counting and flow cytometry techniques to investigate if the velocity measurement can replace these techniques or is giving additional information about the fermentation process.

Besides monitoring of fermentation processes, the developed system might also be a valuable tool in other fields of microbiological research where motility and velocity of microorganisms are of interest.

## Supporting Information

S1 Dataset Details for the comparison of the specific stages of bacterial growth by Mann-Whitney U test. (PDF)

S2 Dataset Raw data of the fermentations 1–3, the shear stress test and the automatic—manual test. (XLSX)

S1 Table Comparison of the specific stages of bacterial growth in different Fermentations by Mann-Whitney U test. (DOCX)
