# Supplementary material for: Single Bacteria Movement Tracking by Online Microscopy – A Proof of Concept Study
Source: PLoS One. 2015 Apr 7;10(4):e0122531. doi: 10.1371/journal.pone.0122531 (PMC4388530; doi:10.1371/journal.pone.0122531)

Oneway Analysis of Data By Typ Fermentation=1

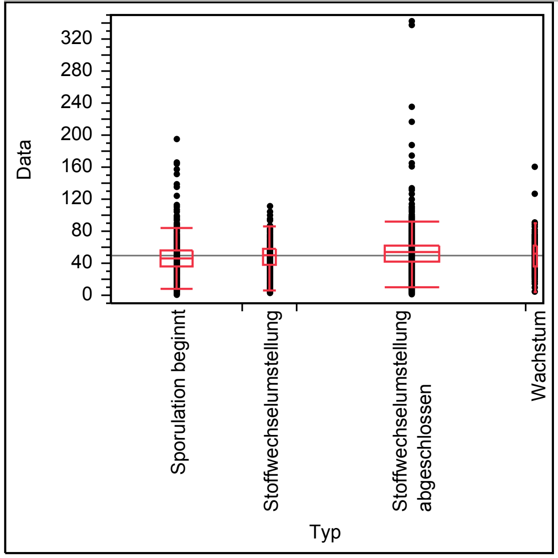

Quantiles

Level	Minimum	10%	25%	Median	75%	90%	Maximum
Sporulation beginnt	0.470086	24.69195	35.93963	46.47785	55.3045	64.1654	195.087
Stoffwechselumstellung	2.85662	24.10542	37.44445	49.2874	58.87315	66.64236	111.325
Stoffwechselumstellung abgeschlossen	0.28406	28.89965	41.51965	53.3775	62.17183	70.21	342.323
Wachstum	4.5015	26.98631	36.80198	52.20115	62.65465	71.61692	160.457

Nonparametric Comparisons For Each Pair Using Wilcoxon Method

q*	Alpha
1.95996	0.05

Level	- Level	Score Mean Difference	Std Err Dif	Z	p-Value	Hodges-Lehmann	Lower CL	Upper CL
Stoffwechselumstellung abgeschlossen	Sporulation beginnt	503.243	39.96702	12.5915	<.0001 *	6.31890	5.36810	7.272600
Stoffwechselumstellung abgeschlossen	Stoffwechselumstellung	236.052	43.26506	5.4559	<.0001 *	3.74910	2.41320	5.090800
Wachstum	Sporulation beginnt	142.180	38.83507	3.6611	0.0003 *	4.55345	2.14260	6.989400
Stoffwechselumstellung	Sporulation beginnt	107.198	30.40499	3.5257	0.0004 *	2.54520	1.14180	3.955800
Wachstum	Stoffwechselumstellung	32.042	20.15947	1.5894	0.1120	2.11440	-0.49760	4.767100
Wachstum	Stoffwechselumstellung abgeschlossen	-82.762	62.57643	-1.3226	0.1860	-1.62200	-4.00430	0.781500

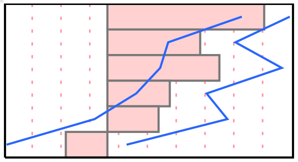

Oneway Analysis of Data By Typ Fermentation=2

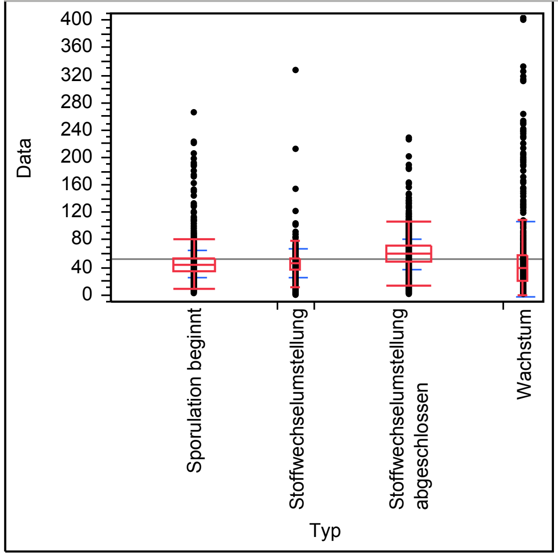

Quantiles

Level	Minimum	10%	25%	Median	75%	90%	Maximum
Sporulation beginnt	1.36826	23.5749	34.51295	44.357	52.77173	62.2307	265.989
Stoffwechselumstellung	0.040274	26.18282	37.06773	46.4287	54.06768	60.59485	327.569
Stoffwechselumstellung abgeschlossen	1.30041	31.19419	47.8249	60.51695	71.28343	83.49631	229.065
Wachstum	0.212621	3.276552	21.176	40.0083	58.2501	97.85134	403.633

Nonparametric Comparisons For Each Pair Using Wilcoxon Method

q*	Alpha
1.95996	0.05

Level	- Level	Score Mean Difference	Std Err Dif	Z	p-Value	Hodges-Lehmann	Lower CL	Upper CL
Stoffwechselumstellung abgeschlossen	Sporulation beginnt	1410.97	44.72994	31.5442	<.0001 *	15.8644	14.9964	16.7334
Stoffwechselumstellung abgeschlossen	Stoffwechselumstellung	868.74	47.87860	18.1446	<.0001 *	14.2708	12.8991	15.6559
Stoffwechselumstellung	Sporulation beginnt	122.40	43.50830	2.8133	0.0049 *	1.7323	0.5263	2.9367
Wachstum	Stoffwechselumstellung	-89.08	20.92178	-4.2575	<.0001 *	-5.1720	-7.5753	-2.8285
Wachstum	Sporulation beginnt	-164.94	42.95496	-3.8397	0.0001 *	-3.7463	-5.6519	-1.8448
Wachstum	Stoffwechselumstellung abgeschlossen	-766.20	47.18872	-16.2369	<.0001 *	-19.0018	-21.0180	-16.9666

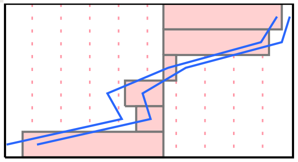

Oneway Analysis of Data By Typ Fermentation=3

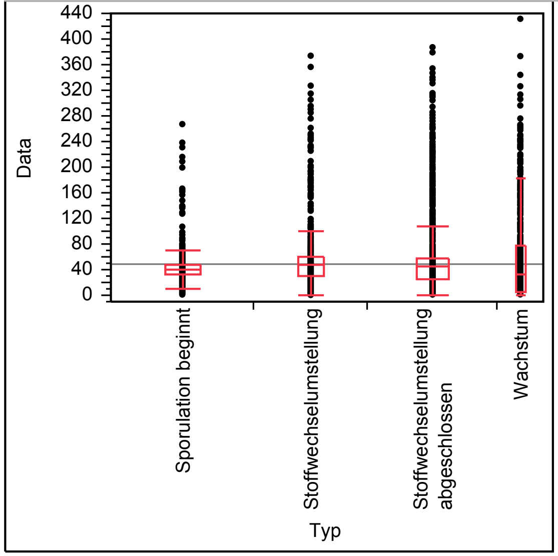

Quantiles

Level	Minimum	10%	25%	Median	75%	90%	Maximum
Sporulation beginnt	0.568646	23.551	32.2754	40.1028	47.2004	54.6236	267.209
Stoffwechselumstellung	0.179848	14.34024	31.0077	46.6994	59.0096	74.95584	373.965
Stoffwechselumstellung abgeschlossen	0.030537	3.443244	25.07745	44.7097	58.19215	96.69828	387.248
Wachstum	0.121151	1.5961	3.79581	33.2116	76.52045	169.5423	431.576

Nonparametric Comparisons For Each Pair Using Wilcoxon Method

q*	Alpha
1.95996	0.05

Level	- Level	Score Mean Difference	Std Err Dif	Z	p-Value	Hodges-Lehmann	Lower CL	Upper CL
Stoffwechselumstellung	Sporulation beginnt	327.547	34.24857	9.56381	<.0001 *	6.00670	4.8048	7.19830
Stoffwechselumstellung abgeschlossen	Sporulation beginnt	200.456	35.08857	5.71286	<.0001 *	3.88370	2.5690	5.17320
Stoffwechselumstellung abgeschlossen	Stoffwechselumstellung	-89.974	33.21866	-2.70855	0.0068 *	-2.19900	-3.8777	-0.59240
Wachstum	Sporulation beginnt	-115.896	33.98477	-3.41024	0.0006 *	-6.51525	-10.3496	-2.77390
Wachstum	Stoffwechselumstellung abgeschlossen	-116.655	32.00443	-3.64497	0.0003 *	-4.95390	-8.9474	-1.87350
Wachstum	Stoffwechselumstellung	-155.105	29.69056	-5.22404	<.0001 *	-9.95995	-13.6890	-5.94760

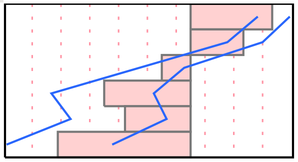

Supplement: S1 Dataset — (PDF) [file pone.0122531.s001.pdf]
