# Supplementary material for: Single Bacteria Movement Tracking by Online Microscopy – A Proof of Concept Study
Source: PLoS One. 2015 Apr 7;10(4):e0122531. doi: 10.1371/journal.pone.0122531 (PMC4388530; doi:10.1371/journal.pone.0122531)
Supplement: S1 Table — (DOCX) [file pone.0122531.s003.docx]

**Table S1.** **Comparison of the specific stages of bacterial growth in different Fermentations by Mann-Whitney U test**

|  | Ferm. 1 vs Ferm. 2 | Ferm. 1 vs Ferm. 3 | Ferm. 2 vs Ferm. 3 |
| --- | --- | --- | --- |
| bevor diauxic shift | <0,0001 | <0,0001 | 0,0235 |
| diauxic shift (min. pH) | 0,0005 | 0,0063 | 0,6976 |
| after diauxic shift | <0,0001 | <0,0001 | <0,0001 |
| start of sporulation | <0,0001 | <0,0001 | <0,0001 |
